# Investigating the Aging Effects of Biochar on Soil C and Si Dissolution and the Interactive Impact on Copper Immobilization

**DOI:** 10.3390/molecules25184319

**Published:** 2020-09-21

**Authors:** Shaojun Jiang, Jiachen Wu, Lianxin Duan, Sheng Cheng, Jian Huang, Tao Chen

**Affiliations:** 1 School of the Environment, South China Normal University, Guangzhou 510006, China; shaojunj93@163.com (S.J.); 15261826395@163.com (J.W.); duanlianxin1993@163.com (L.D.); ShengC@m.scnu.edu.cn (S.C.); 18371807641@163.com (J.H.); 2 Guangdong Provincial Key Laboratory of Chemical Pollution and Environmental Safety & MOE Key Laboratory of Theoretical Chemistry of Environment, South China Normal University, Guangzhou 510006, China

**Keywords:** BC, sequential extraction, copper, soil remediation, carbon-silicon interaction

## Abstract

Aging tests were used to investigate the long-term effects of BC on the immobilization of Cu, and the soil silicon dissolution of three types soils (black soil, (BS), vegetable garden soil (VS) and red soil (RS)). Litchi branch biochars (BC) at 10% (*w*/*w*) were incubated with three Cu (400 mg/kg) contaminated soils. The effect on soil properties of pH, soil organic carbon (SOC), dissolved organic carbon (DOC) and available silicon content were investigated, along with the speciation distribution of Cu. The results indicated that SOC, DOC, and available silicon content (except, BC300) increased with the application of BCs. On the other hand, the DTPA (diethylenetriaminepentaacetic acid) extractable Cu content in BS, VS and RS soils were reduced by 4–12%, 18–25%, and 12–19%, respectively. The Cu availability in all soils first increased, and then decreased during the aging process. The sum of the other four fractions, including the carbonate fraction and the inert component increased by 4–4.5% (BS), 1.4–2.1% (VS), and 0.5–1% (RS) respectively, over the long-term process. Moreover, during the whole aging process, the soil properties (such as pH, SOC, DOC and available silicon content) were almost stable. This study demonstrates that BCs, especially those produced at a higher temperature, are superior to those been produced at 300 °C in immobilizing Cu and releasing available silicon in soils. However, the remediation efficiencies were restricted by the soil type contamination status and remediation time.

## 1. Introduction

Copper pollution in agricultural soils can occur from copper mining and directly or indirectly from anthropogenic sources [1]. This can cause crop yields to fall, as well as allow copper to enter the food chain. Although copper is an essential trace element for the human body, long-term excessive intake can cause copper to accumulate in the body and endanger health [1,2,3,4]. Due to its potential toxicity, persistence and irreversibility by the United States Environmental Protection Agency (USEPA) has listed Cu as priory control pollutant [5,6,7]. With increased public awareness of health and safety, soil contamination by Cu has gained attention, and requires proper remediation.

In-situ remediation of Cu-contaminated soils is regarded as an effective, practicable, and environmentally friendly measure [8]. Biochar (BC) is the solid, carbon (C)-rich and silicon (Si)-rich product of heating biomass in a low oxygen environment (pyrolysis) [9,10,11,12]. Due to its highly porous micro-structure, active functional groups, high pH; rich Si content; surface area; and cation exchange capacity (CEC), BC can effectively immobilize contaminants. It does this by adsorption, ion exchange, surface complexation and precipitation. A number of studies have highlighted that BC further reduces the risk of heavy metals contamination to humans and the surrounding ecosystem [13,14,15,16,17,18]. Recent studies have reported the successful applications of BC in soil remediation, in both the short and long-term. For instance, Rees et al. [19] observed a reduction in extractable cadmium (Cd), copper (Cu (II)), and other metals in soils after the addition of 80% coniferous and 20% hardwood BC after seven days. Uchimiya et al. [20] also found a reduction in Cu (II) and Zn (II), using toxicity characteristic leaching procedure (TCLP) extraction in a contaminated soil 7 day incubation experiment after addition of cottonseed hull BC. Likewise, Bian et al. [21] used wheat straw BC to treat agriculture land and consistently and significantly increased soil pH, total organic carbon and observed reductions in Cd (II) and Pb (II) concentrations, following CaCl_2_ and DTPA extractions over a three year period. Li et al. [22] demonstrated the link between BC type and long-term (three years) immobilization of Cd and Cu in acidic paddy soils, and recommended that the readily oxidized BC be applied to the soil to reduce the risk of Cd and Cu exposure. These short-term and long-term studies demonstrate that the application of BC in soil remediation is feasible. However, the data on long-term effects of BC on the immobilization of heavy metals appear to be inconsistent. In lightly contaminated agriculture soil, Lucchini et al. [23] demonstrated that BC caused small changes in metal fractionation in soil, but no significant changes in total metal concentrations in soil or plants were observed after three years. Over three consecutive harvests, hardwood BC treatments increased the uptake of metals in ryegrass in the last two harvests compared to the first harvest [24]. These studies highlight that the effects of BC on immobilizing heavy metals in soils need to be investigated in greater detail. Furthermore, the long-term immobilization of heavy metals in soils is affected by the aging process of BC [22,25]. BC undergoes a slowed growth rate with uncontrolled in-situ oxidation in soils, which leads to the formation of carboxylic, carbonylic, phenolic and other oxygen-containing functional groups on the surface of aged BC [22,25,26,27,28,29]. As, such, the aging process is not synchronized in soils of different types [27,30,31].

Lots of studies have focused on the organic component of BCs after adding them to soil, because of their function in carbon sequestration and environmental remediation, such as the adsorption of contaminants by oxygen-containing functional groups [12,32,33]. However, the function and effects of BC on the inorganic components are unclear, especially for BC that have higher mineral content (silicon-containing component). On the one hand, BC acts as a potential bio-available Si source and, more importantly, along with Si and Al complexes from soil, results in an increase in Fe and soil organic matter [34]. For instance, the adsorption of silicic acid on Al or Fe oxides may promote a lower phosphate-fixation in acidic soils, thus improving the rate of available P. Silicic acid dissolved in the soil solution can be adsorbed into soil minerals, particularly Fe and Al oxides/hydroxides [35]. There is a general lack of knowledge about the effects of BC aging on silicon flow and pollutant control in soil systems. Silicon-rich biochar is considered a rich source of silicon materials [36]. Therefore, BC can be redefined as a silicon-improving material in soil. Liu et al. [37] reported that the effect of wheat straw BC on soil Si in different regions. Unfortunately, the interaction between soil-available Si content and carbon components with biochar during the aging process in soils was not fully understood. In addition, the stable organo-mineral (C and Si) fraction produced via the aging process between BC and minerals plays a vital role in the stabilization of heavy metals.

Based on the expounded data, we propose the hypothesis that the interaction between soil-available Si content and carbon components in biochar during the aging process assists Cu immobilization immediately following biochar amendment. To test this hypothesis, this study was set up to explore the relationship between C and Si in soil, as well as to determine the availability of Cu in three different soils, during the aging process. The oxidizability under accelerated aging of BC under wet-dry conditions was simulated in the laboratory to gain insights into the aging process of BC. Specifically, this study aims to: (1) compare the effect of BC under different aging processes on three soil properties, and the mobility Cu in soil over the course of a year, and (2) determine the relationship between C and Si in the soil, with added BC, and as a result of stimulated aging.

## 2. Materials and Methods

### 2.1. Soils and Biochar

Three topsoil samples (0–20 cm) were collected from different site fields. one sample of black soil (BS) was collected from non-polluted agricultural fields near Harbin, China (45°40′ N and 126°35′ E); Two samples of red soil (RS) and vegetable garden soil (VS) were collected from agricultural fields (23°07′ N and 113°43′ E) and vegetable fields (23°02′ N and 113°43′ E) in Guangzhou, China. The sample of RS and VS were classified as clay soil (Ultisols), BS was classified as sandy loam soil (Histosols). After collection, the samples were air-dried, and passed through 2 mm and 0.147 mm nylon sieves before using for further characterization analysis.

Litchi branches were collected from an orchard in ZengCheng District, Guangzhou City, Guangdong Province, south China (23°41′ N and 113°81′ E). The BC preparation process followed the method outlined in a previous study [38]. The BCs obtained under oxygen-limited conditions of different temperatures were named BC300 and BC600, where the suffix number refers to the carbonization temperature. The properties of the soils and BC are listed in Tables S1 and S2.

### 2.2. Experimental Design 

The evaluation of retention properties Cu^2+^ on BC300 and BC600, followed the method described by Lin et al. [39]. Briefly, an aliquot of 0.05 g BC sample was added to 50 mL centrifuges tubes (polypropylene) that contained 40 mL of 50 mg/L Cu^2+^ solution and 0.01M NaOH or HCl was used to adjust the solution pH. Then, the tubes were shaken at 250 rpm for 24 h at room temperature (25 °C ± 2) using a horizontal shaker. The amount of metal adsorbed was also calculated following the method described by Lin et al. [39].

The soil was air-dried and sieved to remove impurities, such as stones and organic residues. The total Cu content was adjusted to 400 mg/kg by adding Cu(NO_3_)_2_ solutions, mixed thoroughly, and aged at room temperature for 1.5 months. This study included nine treatments: BS0, VS0 and RS0 (control, containing BS, VS and RS without BC amendment), BS1, VS1 and RS1 (containing, BS, VS and RS with 10% (*w*/*w*) BC300 amendment), BS2, VS2 and RS2 (containing, BS, VS and RS with 10% (*w*/*w*) BC600 amendment). All treatments were performed in triplicate. The incubation experiment was conducted in a constant temperature room at 25 °C with 65% relative humidity, under a wet and dry (wet-dry) cycle for one year. The samples were checked regularly, and water added to compensate for water loss every 10 d to maintain 80% water holding capacity (WHC), then air-dried in the nature environment. During the incubation, soil samples (∼50 g) were collected on days 0, 1, 3, 5, 7, 14, 21, 28, 60, 180 and 365 from each pot after thoroughly mixing the soils. Soil samples were air-dried and sieved for further analysis.

### 2.3. Chemical Analysis

Soil properties, including pH, organic matter, cation exchange capacity, available nitrogen and phosphorus contents and other physicochemical properties, were determined according to previously described methods [40]. Briefly, the pH (1:2.5 of soil:water), soil organic carbon (SOC) (K_2_CrO_4_-H_2_SO_4_ oil-bath heating), cation exchange capacity (CEC) (1 M ammonium acetate leaching at pH 7.0), total N (TN) (N/C soil analyzer, Flash, EA, Milan, Italy), available P (AP) (Olsen method) and available K (AK) (1 M ammonium acetate extraction) were determined. The dissolved organic carbon (DOC) was determined extraction of the soil (5 g) with distilled water (25 mL) and shaking for 2 h. The extract solution was filtered (0.45 μm) prior to DOC determination using a TOC analyzer (TOC V-CPH, Jena, Germany). The available Si (acetate buffer method) from soil using the method adopted from Yu et al. [41]. The total Cu, and plant-available Cu of soil were estimated according to the method by Lu [42]. Briefly, the total heavy metals in the soil were digested by HNO_3_-HF-H_2_O_2_ and the plant-available Cu of soils were extracted from the treated soil using diethylenetriaminepentaacetic acid (DTPA, pH 7.3). The different forms of Cu metal in soil were extracted following the method of Tessier [43], and the methods are summarized in Table S3. All samples were centrifuged at 3000 rpm for 5 min, then, the suspensions were filtered through a 0.45 μm filter, acidified (with concentrated HNO_3_) and Cu concentrations were determined using an atomic absorption spectrophotometer (AAS, iCE 3500, Thermo Scientific, MA, USA).

### 2.4. Data and Statistical Analysis

In the study, the following parameters were evaluated:

(1) Effects of BC application on an increase or decrease in Si or DOC content in soil calculated by Equation (1):

(1) $$E_{\mathrm{bc}}=\frac{\left[\mathrm{Si}\;\mathrm{content}\;\mathrm{or}\;\mathrm{DOC}\;\mathrm{content}\;\mathrm{in}\;\mathrm{soil}\;\mathrm{from}\;\mathrm{objects}\;\mathrm{with}\;\mathrm{application}\;\mathrm{of}\;\mathrm{BC}\right]}{\left[\mathrm{Si}\;\mathrm{content}\;\mathrm{or}\;\mathrm{DOC}\;\mathrm{content}\;\mathrm{in}\;\mathrm{soil}\;\mathrm{from}\;\mathrm{objects}\;\mathrm{without}\;\mathrm{the}\;\mathrm{application}\;\mathrm{of}\;\mathrm{BC}\right]}\times 100\%$$

where, E_bc_—percent increase or decrease in Si content or DOC content in soil under amendment with BC.

(2) The immobilization effectiveness (IE) of Cu in soil was calculated by Equation (2):

(2) $$\mathrm{IE}\left(\mathrm{Cu}\right)=\left(1-\frac{C_{t}}{C_{o}}\right)\;\times \;100\%$$

where, C_t_ and C_o_ are the copper concentration in the leachate of the treated soil and control, respectively.

All the experiments were conducted in triplicate. Data are presented as means with standard deviations. Statistical analyses were performed using SPSS Statistics 19.0 software (IBM, Armonk, NY, USA). A one-way ANOVA with multiple comparisons by LSD test at *p* < 0.05 significance level was used to determine differences. Graphs were prepared using Origin 9.0 (OriginLab Corp, Northampton, MA, USA).

## 3. Results and Discussion

### 3.1. Soil Properties of Soil and BC

The soil properties are summarized in Table S1. Analysis of the soil properties and the pH shows that there were differences among the collected soil samples. The pH values followed the order BS (pH = 6.15) > VS (pH = 5.88) > RS (pH = 4.86). Likewise, the highest and lowest SOC were observed in BS and RS, respectively, BS and RS were collected from north and south China, respectively, indicating the positive correlation between latitudes for different sites. Moreover, they had the highest extractable cation contents of available silicon and BS compared with the other soil samples. However, the lowest content of available silicon and low CEC values were observed in RS and VS. Prior to spiking with Cu, these three soils were considered unpolluted, because the concentration of heavy metals, such as Cu, Pb, Cd and Zn concentration in soils all lower than the risk screening values for soil contamination of agricultural land (Table S1) [39]. In comparison, the DOC of BCs gradually decreased due increased degradation of carbon components at high temperatures. Noticeable, as shown in Table S2, the C content in BC gradually increases with the pyrolysis temperature increases, while the H, O, and N content continues to decrease, and the atomic ratios H/C, O/C, and (O + N)/C also decrease. These results indicate that biochar with a higher pyrolysis temperature has a higher degree of carbonization and a more complete π-conjugated aromatic structure [39]. Litchi BC morphology and the absorbent capacities have been discussed in the literature [38,39,44]: the observed increase in the p-conjugated system, and metal-retention capability. Therefore, this was not considered in the current study.

### 3.2. Sorption Behavior of Cu by BCs

**Figure 1:**
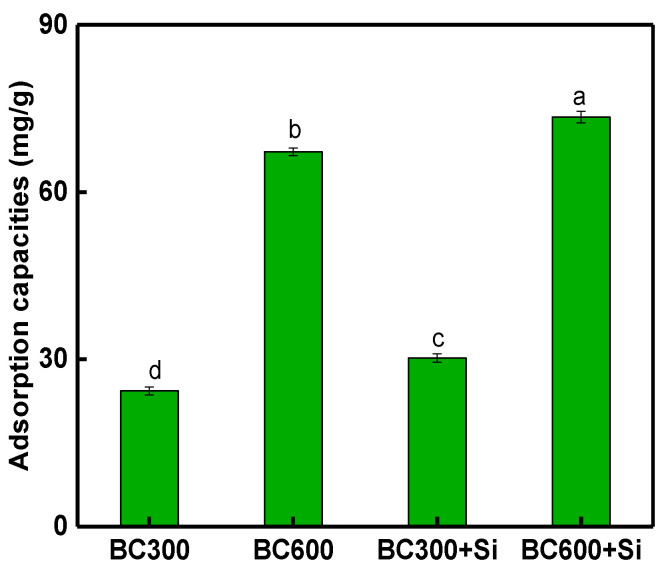
The adsorption capacities of Cu on different BC. BC300 + Si and BC600 + Si are BC modified with 5 M sodium silicate. Values are the mean ± standard deviations, different letters in the same column represent significant difference at *p* < 0.05 (*n* = 3, LSD test).

The sorption capacities of Cu, by different BCs are presented in Figure 1. The maximum adsorption capacities for Cu increased with the rising pyrolysis temperature from 24.33 (for BC300) to 67.23 mg/g (for BC600), respectively. Notably, the maximum adsorption capacity between BCs of different temperatures different higher than previous studies (Table S4). This can be attributed to the pH, ash contents, functional groups and high CEC values of the BCs (Table S1). This result is consistent with other studies [45] and indicates the positive influence of BC inorganic compounds on metal immobilization in aqueous solutions. In addition, an amount of studies on the large surface area, pore structure and functional group of biochars indicated that metal immobilization was possible [12,13,22]. Interestingly, following BC treatment with sodium silicate, the Cu sorption capacities in BC300 + Si and BC600 + Si increased by 24% and 9%, respectively, compared to BC300 and BC600. Zhao et al. [46] from an adsorption study suggested that the addition of silicon not only enhanced the adsorption capacity of biochar, but also effectively resisted the decrease in the adsorption capacities on BCs after aging (H_2_O_2_ and H_2_SO_4_ oxidation). Previous studies have also shown that silica particles might affect C arrangement and structural composition by producing a Si-C coupling system in the biochar matrix [47]. As discussed above, BC modified with silicon shows potential for enhanced metal adsorption.

### 3.3. Effects of BC Amendment on Soil pH, DOC, and SOC

**Figure 2:**
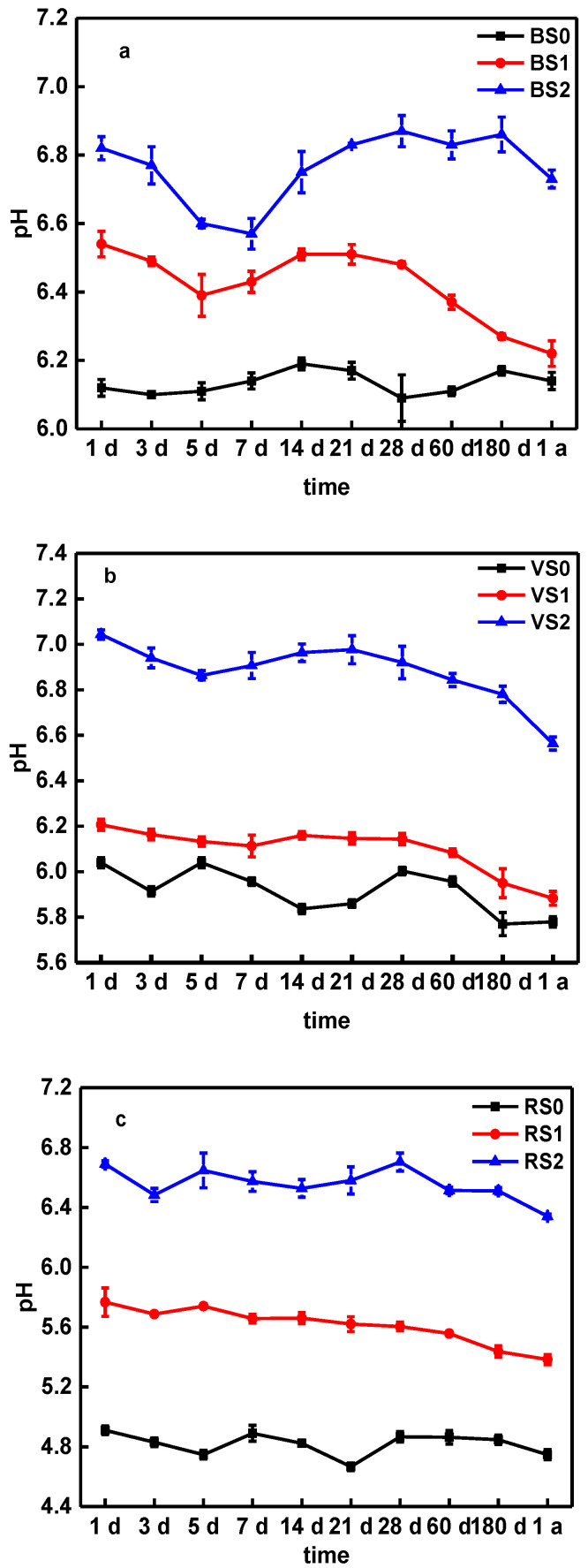
The change of pH in BS (**a**), VS (**b**) and RS (**c**) soils amended with biochars. Note: Error bars are standard deviations of the means (*n* = 3). (BS0: black soil, BS1: black soil + BC300, BS2: black soil + BC600, VS0: Vegetable garden soil, VS1: Vegetable garden soil + BC300, VS2: Vegetable garden soil + BC600, RS0, Red soil, RS1: Red soil + BC300, RS2: Red soil + BC600).

**Figure 3:**
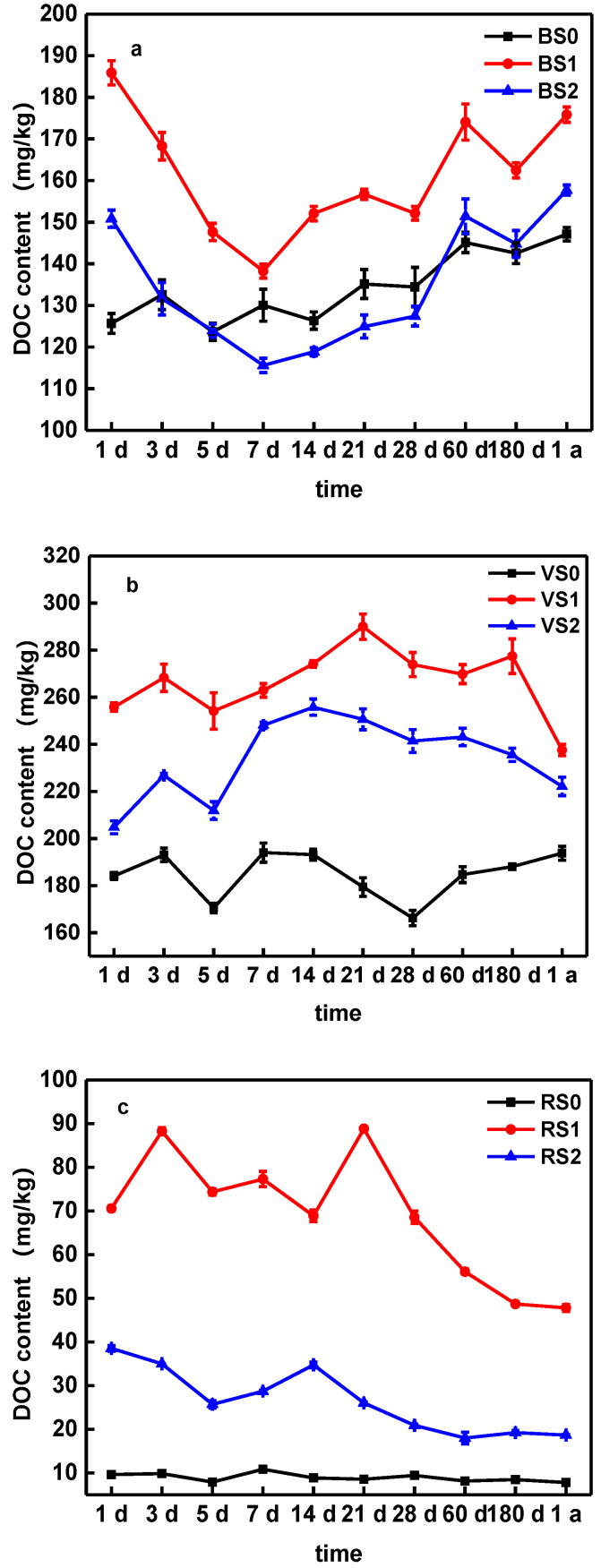
The change in DOC content in BS (**a**), VS (**b**) and RS (**c**) soils amended with biochars. Note: Error bars are standard deviations of the means (*n* = 3).

The impact of BC on soil pH, DOC and SOC, over one year is shown in Figure 2 and Figure 3, Table 1 and Table S5. Compared with the control, the application of BC slightly elevated soil pH, and the influence was more obvious with the application of biochar that was higher in pH (Figure 2). The pH values of BS0, VS0 and RS0 showed a slow, slightly increasing trend at certain time points, during the whole incubation process, which may be related to the neutrality of the deionized water used for wet-dry cycle (Figure 2). After BC treatment and aging, the pH of BS1 and BS2 on day 5 decreased to 6.39 and 6.60 compared with the pH on day 1, and rapidly increase to 6.51 and 6.87, from day 3 to day 28, followed by a slow decrease during the whole aging process (Figure 2a).

However, compared to BS the pH changes of VS and RS were different during aging process, mainly related to differences in soil types and properties. After biochar treatment and aging, the pH of VS1, VS2, RS1 and RS2 rapidly increased on day 1, then slowly decrease during the by one-year aging process (Figure 2b,c). Previous studies have shown that BC during the 72-day aging process had the effect of improving the soil pH, particularly in the early stage [48]. Wang et al. [33] showed that pH rapidly decreased from 0 to 30 days and slowly decreased from 31 to 95 days. Xu et al. [49] showed that pH (BC) value decreased from 8.20–10.7 to 7.5–9.7 after a 25-cycle aging test with BC, which can explain the soil slow decreased for pH during aging process (Figure 2).Furthermore, in a field experiment under a cucumber–sweet potato–rape rotation, Jiang et al. [44] showed that BC application can alter the soil acidity, while the natural aging of biochar in soil, cause the pH of soil to slowly decrease.

As SOC did not significantly change during the whole incubation process in any of the same treatments soil, we did not measure SOC at every time points, indicating that both types of biochar remained stable in the soil in the long term [22]. Changes in BC significantly increased the SOC content, compared with the control (Table 1). The increase in SOC was greater among VS and RS, compared to BS. Likewise, in soil treated with BC, DOC was significantly higher than in the untreated soil. The higher the pyrolysis temperature, the weaker the effect of improving soil DOC (Figure 3), which may due to the high DOC in BC300 (Table S1).

The DOC concentrations in the soil, in common with pH, showed variation between collection periods. However, there was consistent decreasing trend in VS and RS in the latter part of the aging process, compared to the early soil (Figure 3b,c). With treatment by BS, however, the DOC content in BS1 and BS2 decreased from 1day to 7 days, and then increased from day 7 until the end of the process. This could be because BC improves the soil environment and enhances the adsorption of soil to organic components in the initial stage, but the labile organic compound in soil and biochar samples may be released during incubation after 7 days (Figure 3a). A number of studies have also reported that BC application could significantly increase DOC content in soils and decreased during long term aging process [24,50,51]. Li et al. [22] showed that the contents of soil organic matter (SOM) and DOC were significantly higher in the soil treated with biochar. There was a slow change in SOM during the 3-year period; DOC contents, on the other hand, changed over time, increasing steadily up to harvest one, and then decreasing over harvest 2 and 3. BC can modify soil DOC, in both quality and quantity by means of releasing DOC into the soil solution and/or stimulating the generation of more DOC from the soil organic carbon pool [51]. Moreover, Jiang et al. [44] reported that there was a downwards trend in DOC content after the aging process of crop cultivation. This may be attributed to the mineralization of C that occurs during the growing season, in the presence of N from inorganic fertilizer.

### 3.4. Effect of Biochar on Soil Available Silicon

**Figure 4:**
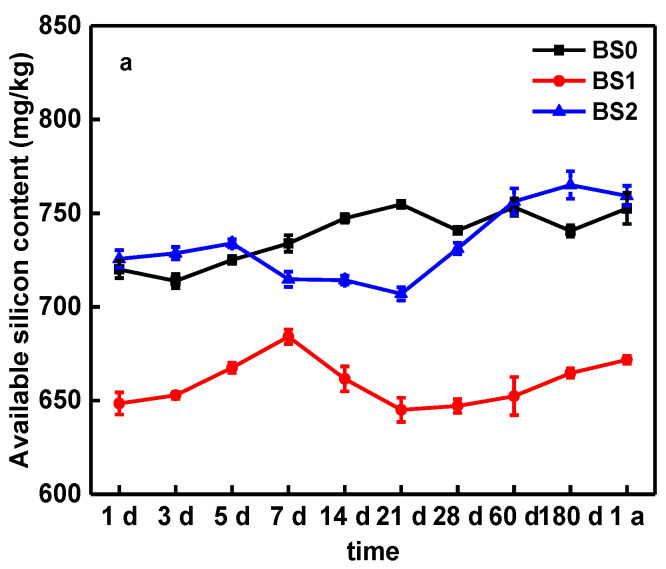

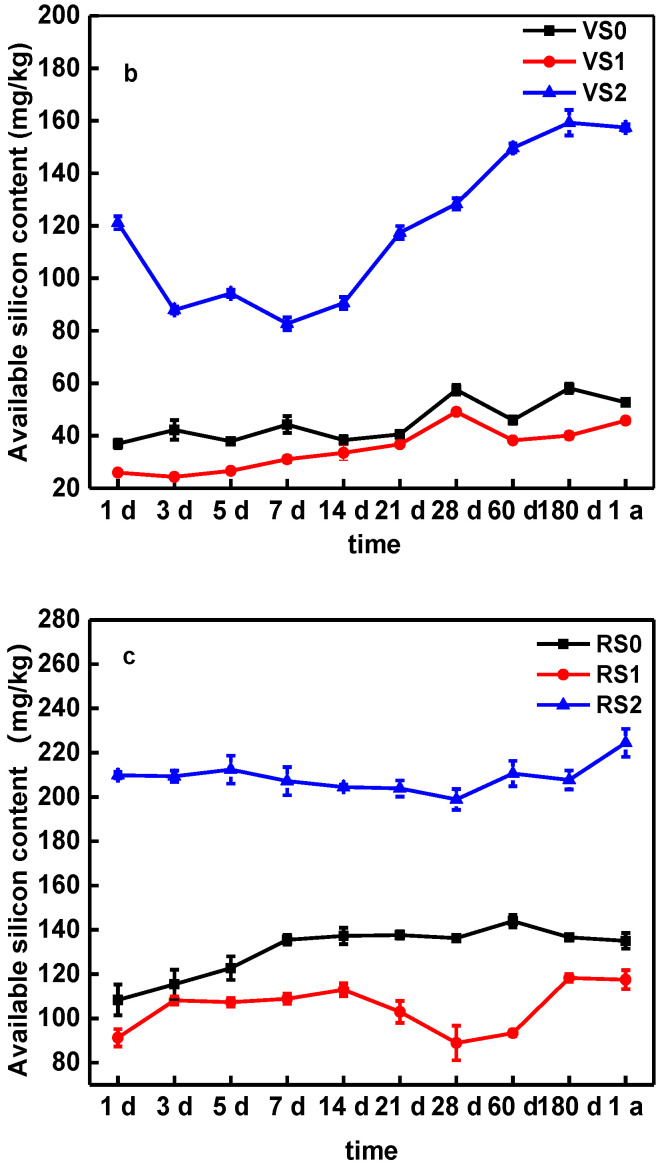
The change of available silicon content in BS (**a**), VS (**b**) and RS (**c**) soils amended with biochars. Note: Error bars are standard deviations of the means (*n* = 3).

Silicon, as a chemical modification in its soluble form, can effectively decrease heavy metal bioavailability [52]. The role of Si in BC, on Cu mobility during the aging process of typical soils, such as BS, VS and RS, is not yet fully understood. The results of the concentrations of available Si in soil amended with BCs are shown in Figure 4. Figure 4 a show that BS1 decreased the available silicon by 11% and 12% compared with BS0 and BS2 in last stage, respectively. Due to the increase in available silicon content in the later stage of the aging process, BS2 is close to BS0 in the final stage. During the entire aging process, the available silicon content in BS1 was always lower than BS0.This is due to the BC300 having lower ash and SiO_2_ content (Table S1) and potentially an equilibrium has been reached between the Si adsorbed by soil and the Si dissolved from BC surface [32]. Compared with VS0 and RS0, the treatments VS1 and RS1 also show the same trend during o1a aging process (Figure 4b,c).

Similarly, compared to VS0 and RS0, the available silicon of VS2 and RS2 significantly increased by 87–228% and 46–94%, respectively, with the application of BC600 (Table S5). This result is due to the fact that Si the major component of the ash and BC derived from BC600 had a higher Si content, thus resulting in enhanced Si available for the lower Si soils, such as VS and RS. Previous studies have shown that BC can be used as a Si pool. It supports the dynamic process of Si release from BC modified soil [11,12,32,52].

Figure 4 also shows the silicon dissolution of the experimental values for different soil-BC mixtures. For BC600, the higher improvement in silicon dissolution in a low-silicon soil (RS) compared to that in a high-silicon soil (VS) treated with Si-rich BC (BC600) was observed. However, the opposite result was reported by Wang et al. [32]. For Si-rich BCs, the higher improvement in silicon dissolution in a high-silicon soil (HSS) compared to that in a low-silicon soil treated with Si-rich BCs was observed [32]. According this phenomenon, we suggest that the interaction between the soil and the BC may have caused the change, the BC and soil properties major the influence factor.

### 3.5. DTPA-Extractable Cu in the Soil

**Figure 5:**
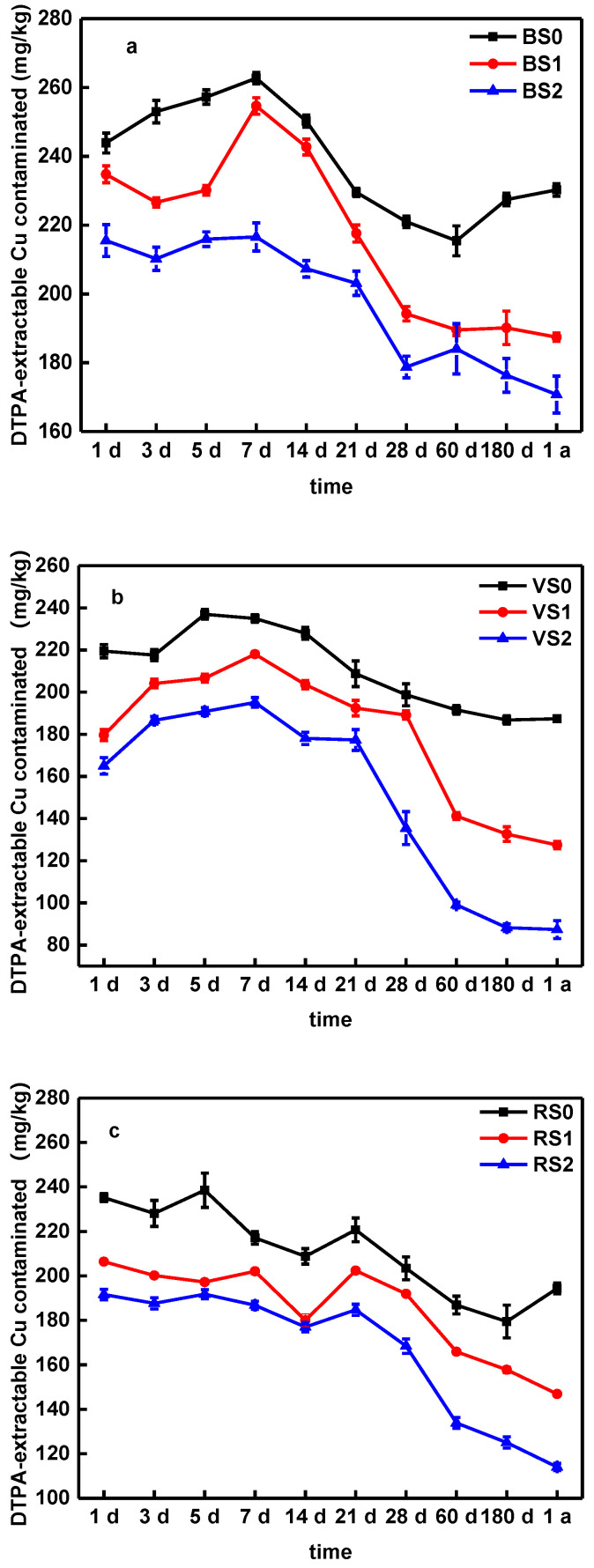
The change of DTPA-extractable Cu concentration in BS (**a**), VS (**b**) and RS (**c**) soils amended with biochars. Note: Error bars are standard deviations of the means (*n* = 3).

As shown in Figure 5, BC amendment reduced the mobility of Cu contents in soils, as seen by the reduction of DTPA-extractable metal content. By comparison, BC600 was more effective at metal immobilization than BC300, and achieved the highest reduction in the DTPA-extractable metal content in the three soils. This is because the adsorption capacities were higher than other BCs (Table 2). In addition, a clear difference was found between treatments with different soils. Xiao et al. [45] showed that BCs were more effective at reducing DTPA-extractable metal contents and metal immobilization than straw materials, this led them to believe that adsorption might not be the only mechanism governing metal immobilization in soils. Similarly, He et al. [53] demonstrated that the BC soil oxidation reaction would significantly affect the performance of BC on metal mobility and speciation in soils. Compared to BS, this result indicates that the BC (BC300 and BC600) significantly affects the immobilization of Cu in RS and VS. The Cu immobilization rates were higher in BS during the one-year aging process, which might be due to a lower concentration of available Cu in VS and RS, and because the bond between Cu and soil in BS was not as strong (Figure 5 and Table 2).

Beesley and Dickinson [54] showed that the application of hardwood-derived BC increased the concentrations of DOC, which has an adverse effect on the immobilization of Cu. Therefore, the DTPA-extractable Cu contents higher than other soils during one-year aging process is probably due to the increase in DOC (Figure 3) and SOC (Table S1) in the BS treatments (BS0, BS1 and BS2). In terms of the change trends, the Cu availability in nine treatments fluctuated sight, but generally showed an increase-decrease trend. For BS, the DTPA-extractable Cu concentration decreased from 215 mg/kg on day 1 to 170 mg/kg on one year with BC600 application (Figure 5a). The DTPA-extractable Cu concentration in BS1 fluctuated between 187 and 234 mg/kg, accounting for 47–58% of the BS total Cu on the incubation (Figure 5a). Compared to BS, the DTPA-extractable Cu concentration in VS and RS also have be similar on trend during one-year aging process (Figure 5). The changes in Cu availability in soils reflected that the transport and transformation processes between soil and BC were dynamic during the aging process.

Taking into consideration the pH value (Figure 2), DOC (Figure 3) and available silicon content (Figure 4), we suggest that the interaction between these factors and the soil DTPA-extractable Cu may have caused the change in Cu availability. Previous works [22,24,45,52] have also shown that an increase in soil pH, DOC and Si content after BC application could reduce the mobility of metal and promote its transformation to a more stable state. The aging experiment reported by Shen et al. [17] showed that the pH of the biochar decreased after accelerated aging due to the loss of dissolved alkaline minerals, indicating the pH could be a mobilization mechanism of the Cu during accelerated aging. Li et al. [51] study showed that a BC induced DOC modification in soil alters the speciation of metal contaminants by changing soil conditions, which suggests that the biochar-DOM interaction is an important mechanism in determining the mobility of heavy metal contaminants in biochar amended soils. Moreover, silicon from BC, as a chemical amendment in its soluble form, can effectively decrease heavy metal bioavailability [52]. Therefore, the pH values of soil, DOC, available silicon content and DTPA-extractable Cu concentration are dynamic during the aging process with BC application, indicating there was a close relationship between them.

### 3.6. Tessier Cu Fractions in the Soil

**Figure 6:**
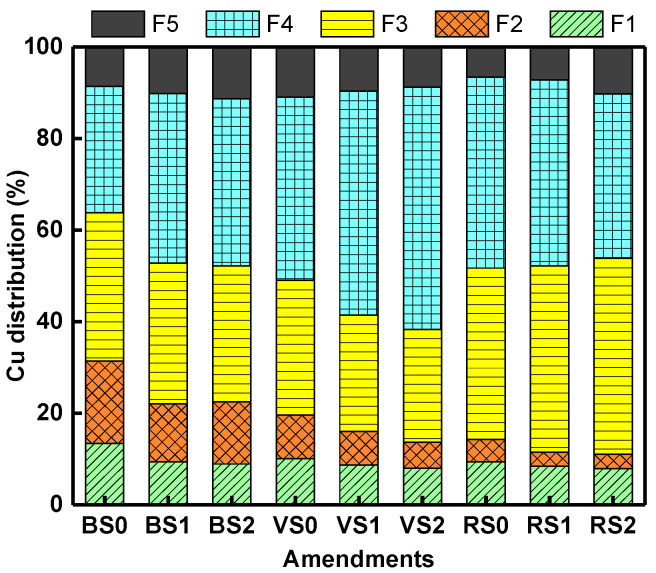
Cu fractionations of soils after 1a incubation with different BC amendments. F1, exchangeable; F2, carbonate bound; F3, Fe-Mn oxide bound; F4, organic matter bound; F5, residual fraction.

The Tessier method was used to determine the five Cu fractions in the soil. The five Cu fractions in the soil were significantly affected in all nine-soil treatments during one-year aging process, compared with the early aging stage (i.e., on day 1). However, a slight change occurred at each time points: in general, F1 and the sum of F1 and F2 showed decreasing trends. Hence in this study, we only evaluated and compared the five Cu fractions at the last stage (i.e., at one year). Figure 6 shows the fractionation of Cu in different treatments. We can see that the Cu bound to Fe and Mn oxide (F3), organic matter (F4) and the residual fraction (F5) covers over 68–89% of its total content in almost all the treatments.

Comparing the three soils, F1 and the sum of F1 and F2 is higher in BS than in VS and RS. This also supports the high DTPA-extractable Cu concentration for BS (Figure 6). The sum of F1 and F2 of BS0, BS1 and BS2 were 31%, 22% and 23%, respectively, and higher than the VS and RS treatments; The phenomenon might occur because SOC (Table S1) and DOC (Figure 4) is high in the BS, both of which decreased the ability of BC to fixate Cu [54]. Although, Park et al. [10] reported that Cu is present as a more stable fraction complex in this soil, but we believe that Cu in artificially contaminated soil more easily complexes with DOC in the soil during the one-year aging, enabling high SOC and DOC content. It is well known that Pb in F3, F4, and F5 is an inert component. While, easily exchangeable Cu (F1) is directly related to its bioavailability, and Cu bound to carbonates (F2) can be converted to bioavailable component as soil pH decreases. Meng et al. [48] indicated that BC continuously immobilized the bioavailable metal fraction in the soil during simulation of the actual aging process over 8 years. Therefore, the sum of the other four fractions including the carbonate fraction, F3, F4 and F5 all significantly increased in the long-term process.

### 3.7. Limitation and Environmental Implication

As demonstrated above, biochar is capable of immobilizing Cu in soils. However, soil properties, such as pH, SOC and DOC, and the properties of BC and the aging time, all affect the of efficiency of metal immobilization. As shown in Figure 2, the application of BC facilitated the stabilization of pH in the soil, but in the long-term aging process, the soil pH decreased. Therefore, in order to maintain the long-term immobilization of Cu in soils by BC, it is appropriate to add new BC in the long-term amended stages of soil. BC also plays a significant role in various terrestrial biogeochemical processes of Si and C (Figure 3 and Figure 4): however, the Si content in BC for amended pollution has not caused issues. In this study, we first considered the available silicon content, in relation to Cu immobilization during aging process. The results show that the release of available Si is important to the fixation of Cu, which is consistent with the experimental results (Figure 1).

Moreover, the effect of BC on soil Si in different soils was investigated, our results were contradictory to other studies, e.g., Wang et al. [32] with regards to whether the available Si content of soil increased or decreased after BC amendment. This may be due to feedstocks of BC and soil properties. More studies are needed to focus on the carbon-silicon biogeochemical cycles and the heavy metal transport distribution between soil and water ecosystems. On the other hand, our result indicate that BC continuously immobilized the bioavailable Cu fraction in the soil (Figure 5 and Figure 6),and BC has been applied to heavy metal contaminated soils as a metal stabilizing method, but it is unclear when and in what form the fixed heavy metals are released again. Further studies should focus on combining the Si source with the heavy metal (BC or soil) in contaminated soil during the aging process.

## 4. Conclusions

In this study, the addition of BC facilitated the immobilization of Cu in three soils types. However, the efficiency was affected by both the properties of the BC and the incubation time, as well as soil type. BC may not perform well in Cu contaminated soils with high SOC and DOC content. In comparison, the addition of BC enhanced soil DOC and pH value in VS and RS and significantly decreased the available Si content in both BS1 and BS2 during the aging process. Moreover, all the treatments significantly increased the SOC content. Generally, BCs, especially those high in pH and high ash content, had a better performance on soil Cu immobilization, and BC application facilitated the transformation of Cu speciation from F1 or F2 into a stable fraction. Although BC application reduced the extractable Cu contents in DTPA-extracts, the remediation performance was not satisfactory, especially for BS soil treatment due to the high DTPA-extractable Cu concentration during the whole aging process. This study showed that field applications of BC in heavy-metal associated soil remediation must consider the soil type and region attributes into account, and a study with respect to the soil type and the Si source (BC or soil) in contaminated soil during the aging process are important when BC applications are evaluated.

## Figures and Tables

**Table 1:** Soil organic carbon (SOC), as affected by biochar (BC) application after one-year of aging. BS, black soil. VS, Vegetable garden soil. RS, red soil.

Treatment	BS0	BS1	BS2	VS0	VS1	VS2	RS0	RS1	RS2
SOC (%)	3.32	5.07	4.38	1.53	3.27	2.37	0.90	2.41	1.85

**Table 2:** The immobilization effectiveness (IE) of Cu in three amended soils with biochars (BCs). Values are the mean ± standard deviations, and the different letters in the same column represent that significant difference at *p* < 0.05 (*n* = 3, LSD test).

	Time	BS		VS		RS	
		BC300	BC600	BC300	BC600	BC300	BC600
IE(Cu,%)	1 d	4 ± 0.25 f	12 ± 0.85 h	18 ± 0.17 d	25 ± 0.53 d	12 ± 0.29 d	19 ± 0.24 e
	3 d	10 ± 0.53 d	17 ± 0.66 f,g	6 ± 0.54 i	14 ± 0.57 g	12 ± 0.37 d	18 ± 0.37 e,f
	5 d	11 ± 0.33 d	16 ± 0.33 f,g	13 ± 0.16 e	19 ± 0.33 f	17 ± 0.41 b	20 ± 0.76 d
	7 d	3 ± 0.21 g	18 ± 0.57 d,e	7 ± 0.21 h	17 ± 0.16 g	7 ± 0.34 e	14 ± 0.54 i
	14 d	3 ± 0.08 g	17 ± 0.49 e,f	11 ± 0.22 f	22 ± 0.24 e	14 ± 0.49 c	15 ± 0.37 h,i
	21 d	5 ± 0.29 e	12 ± 0.24 h	8 ± 0.47 g	15 ± 0.49 i	8 ± 0.29 e	16 ± 0.24 g,h
	28 d	12 ± 0.22 c	19 ± 0.57 c,d	5 ± 0.21 j	32 ± 0.21 c	6 ± 0.17 f	17 ± 0.24 f,g
	60 d	12 ± 0.25 c	15 ± 0.93 g	26 ± 0.53 c	48 ± 0.33 b	11 ± 0.29 d	28 ± 0.33 c
	180 d	16 ± 0.22 b	22 ± 0.24 b	29 ± 0.45 b	53 ± 0.08 a	12 ± 0.17 d	30 ± 0.54 b
	1 a	19 ± 0.12 a	26 ± 0.41 a	32 ± 0.39 a	53 ± 0.49 a	24 ± 0.37 a	41 ± 0.76 a

## Supplementary Materials

The following are available online, Figure S1: The locations of the soil sampling sites, Table S1: The properties of the soil and BC, Table S2: Elemental compositions and atomic ratios of biochars, Table S3: Extractants used in sequential extraction of metal in soil, Table S4 Comparison of sorption capacity of Cu with biochars derived from different materials, Table S5: Effects of the application of BC on an increase/decrease in silicon content/DOC content in soil.
