# Supplementary material for: Investigating the Aging Effects of Biochar on Soil C and Si Dissolution and the Interactive Impact on Copper Immobilization
Source: Molecules. 2020 Sep 21;25(18):4319. doi: 10.3390/molecules25184319 (PMC7570456; doi:10.3390/molecules25184319)
Supplement: Supplementary file 1 [file molecules-25-04319-s001.pdf]

Investigating the Aging Effects of Biochar on Soil C and Si Dissolution and the Interactive Impact on Copper Immobilization

Shaojun Jiang [1,2], Jiachen Wu [1], Lianxin Duan [1,2], Sheng Cheng [1,2], Jian Huang [1,2] and Tao Chen [1,2,*]

[1] School of the Environment, South China Normal University, Guangzhou 510006, China: shaojunj93@163.com (S.J.); 15261826395@163.com (J.W.); duanlianxin1993@163.com (L.D.); ShengC@m.scnu.edu.cn (S.C.); 18371807641@163.com (J.H.)

[2] Guangdong Provincial Key Laboratory of Chemical Pollution and Environmental Safety & MOE Key Laboratory of Theoretical Chemistry of Environment, South China Normal University, Guangzhou 510006, China

* Correspondence: tao.chen@m.scnu.edu.cn; Tel./Fax: +86-20-39310187

Table and Figure captains:

1. *Table S1 The properties of the soil and BC*
2. *Table S2 Elemental compositions and atomic ratios of biochars*
3. *Table S3 Extractants used in sequential extraction of metal in soil*
4. *Table S4 Comparison of sorption capacity of Cu with biochars derived from different materials*
5. *Table S5 Effects of the application of BC on an increase/decrease in silicon content/ DOC content in soil.*
6. *Fig.S1 The locations of the soil sampling sites.*

Table S1. The properties of the soil and BC.

Parameters	Soil			BC	
	**BS**	**VS**	**RS**	**BC300**	**BC600**
pH	6.15	5.88	4.86	7.54	9.53
Soil organic matter (g/kg)	34.2	16.7	9.2	-	-
Cation exchange capacity (cmol/kg)	48.3	20.9	14.2	18.67	26.25
Dissolved organic carbon (mg/kg)	121	199	7.1	738	257
Available silicon (1:25 citric acid, mg/kg)	715	36	105	-	-
SiO_2 (%)	-	-	-	4.70	8.54
Ash (%)	-	-	-	4.88	9.50
S_{BET} (m^2/g)	-	-	-	69.62	183.15
PZC	-	-	-	4.06	5.10
Metal content (mg/kg) Cu	61.1	19.0	8.12	2.12	3.78
Pb	8.13	16.1	7.78	-	-
Cd	< 0.1	< 0.1	< 0.1	-	-
Zn	83.2	45.1	16.7	-	-

BS, black soil.VS, Vegetable garden soil. RS,red soil.

Table S2. Elemental compositions and atomic ratios of biochars.

Biochar	C/%	H/%	O/%	N/%	H/C	O/C	(O+N)/C
BC300	57.13	3.95	22.36	2.46	0.07	0.39	0.43
BC600	74.57	1.51	13.91	3.19	0.02	0.19	0.23

Table S3. Extractants used in sequential extraction of metal in soil.

	Phase	Reagent	Time of shaking
Fraction 1 (F1)	Easily exchangeable	1*M* $MgCl_2$(pH 7)	1 h at 25°C
Fraction 2 (F2)	Bound to carbonates	1*M* CH_3COONa/CH_3COOH (pH 5)	5 h at 25°C
Fraction 3 (F3)	Bound to Fe and Mn oxide	0.04 *M* $NH_2OH·HCl$ in 25%(v/v)CH_3COOH(pH 2)	6 h at 96°C
Fraction 4 (F4)	Bound to organic	30% H_2O_2/0.02 *M*HNO_3 (pH 2), followed by 3.2*M* CH_3COONH_4/20%(v/v) HNO_3	2 h at 85°C/3 h at 85°C
Fraction 5 (F5)	Residual	$HNO_3/HCl/HClO_4$	Digestion

Table S4. Comparison of sorption capacity of Cu with biochars derived from different materials.

Heavy metal	Biochar reparation source	Maximal adsorption capacity (mg/g)	Literature resources
Cu	Hardwood	6.79	Chen et al.(2008)
	Cron straw	12.52	Chen et al. (2011)
	Interweed	48.49	Li et al. (2013).
	Miscanthus	15.4	SHIM et al.(2015)
	Cow dung	54.4	Xu et al. (2013)
	Pig manure	88.23	Kolodynska et al.(2012)
	sludge	14.83	Jin et al.(2016)
	Pine	1.47	Jiang et al.(2016)
	Hickory wood	12.30	Wang et al.(2015)
	Apple tree branch	15.85	Wang et al.(2017)
	Sycamore wood	17.44	Wang et al.(2017)

References:

1. Baoliang, C., Dandan, Z., Lizhong, Z., & Xueyou, S. (2008). Sorption characteristics and mechanisms of organic contaminant to carbonaceous biosorbents in aqueous solution. Science China Chemistry, 051(005), 464-472.
2. Chen, X. , Chen, G. , Chen, L. , Chen, Y. , Lehmann, J. , & Mcbride, M. B. , et al. (2011). Adsorption of copper and zinc by biochars produced from pyrolysis of hardwood and corn straw in aqueous solution. *Bioresource Technology, 102*(19), 8877-8884.

3. Li, M. , Liu, Q. , Guo, L. , Zhang, Y. , Lou, Z. , & Wang, Y. , et al. (2013). Cu(ii) removal from aqueous solution by spartina alterniflora derived biochar. Bioresource Technology, 141, 83-88.
4. SHIM T, YOO J, RYU C, et al. (2015). Effect of steam activation of biochar produced from a giant Miscanthus on copper sorption and toxicity[J]. Bioresource Technology, 197: 85-90.
5. XU X Y, CAO X D, ZHAO L, et al.(2013). Removal of Cu, Zn and Cd from aqueous solutions by the dairy manure-derived biochar[J]. Environmental Science and Pollution Research, 20(1): 358-368.
6. KOŁODYŃSKA D, WNĘTRZAK R. LEAHY J J, et al. (2012). Kinetic and adsorptive characterization of biochar in metal ions removal. Chemical Engineering Journal, 197: 295-305.
7. JIN H M, HANIF M U, CAPAREDA S, et al. (2016).Copper(II) removal potential from aqueous solution by pyrolysis biochar derived from anaerobically digested algae-dairy-manure and effect of KOH activation. Journal of Environmental Chemical Engineering, 2016, 4(1): 365-372．
8. JIANG S S, HUANG L B, NGUYEN T A H, et al.(2016). Copper and zinc adsorption by softwood and hardwood biochars under elevated sulphate-induced salinity and acidic pH conditions[J]. Chemosphere, 142: 64-71．
9. WANG H Y, GAO B, WANG S, et al．Removal of Pb (II), Cu(II), and Cd(II) from aqueous solutions by biochar derived from KMnO4 treated hickory wood[J]. Bioresource Technology, 2015, 197: 356-362.
10. WANG Tong-tong, MA Jiang-bo, QU Dong, et al. （2017）. Characteristics and mechanism of copper adsorption from aqueous solutions on biochar produced from sawdust and apple branch[J].Environmental Science, 38(5):2161-2171.

Table S5. Effects of the application of BC on an increase/decrease in silicon content/ DOC content in soil.

	Time	**BS**		VS		RS	
		**BC300**	**BC600**	BC300	BC600	BC300	BC600
EBC-DOC	1 d	48%	20%	39%	11%	634%	301%
	3 d	27%	-1%	39%	18%	797%	256%
	5 d	19%	-2%	49%	24%	839%	225%
	7 d	6%	-11%	36%	28%	613%	165%
	14 d	20%	-6%	42%	32%	678%	292%
	21 d	16%	-8%	62%	40%	938%	204%
	28 d	13%	-5%	65%	45%	629%	122%
	60 d	20%	4%	46%	32%	590%	121%
	180 d	14%	2%	48%	25%	475%	127%
	1 a	20%	7%	23%	15%	513%	139%
EBC-Available Si	1 d	-10%	1%	-30%	228%	-16%	94%
	3 d	-9%	2%	-42%	108%	-6%	81%
	5 d	-8%	1%	-30%	149%	-13%	73%
	7 d	-7%	-3%	-30%	87%	-20%	53%
	14 d	-11%	-4%	-13%	136%	-18%	49%
	21 d	-14%	-6%	-9%	190%	-25%	48%
	28 d	-13%	-1%	-15%	123%	-35%	46%
	60 d	-13%	2%	-17%	225%	-35%	46%
	180 d	-10%	3%	-31%	174%	-13%	52%
	1 a	-11%	1%	-13%	199%	-13%	66%

BS, black soil.VS, Vegetable garden soil. RS,red soil; EBC-DOC, The percent increase or decrease in DOC content in soil under amendment with BC; EBC-Available Si, The percent increase or decrease in available Si content in soil under amendment with BC

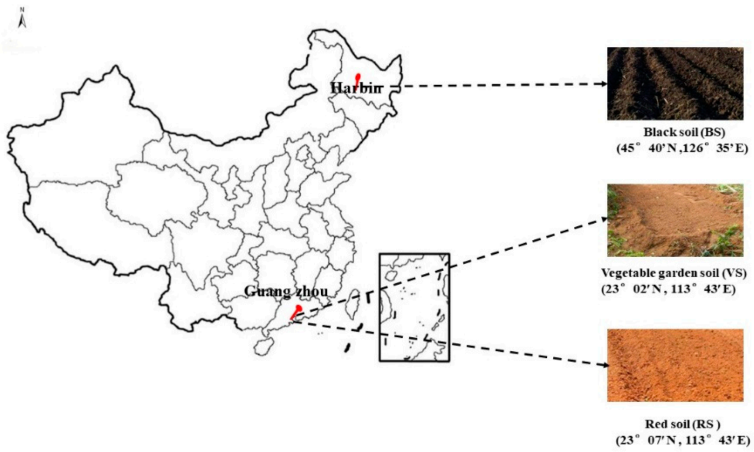

Figure S1. The locations of the soil sampling sites.
